# Exergames for falls prevention in sheltered homes: a feasibility study

**DOI:** 10.3389/fpubh.2024.1344019

**Published:** 2024-06-21

**Authors:** Emma Stanmore, Charlotte Eost-Telling, Wytske Meekes, Kelly Banham, Jay Chillala, Bibhas Roy, Joseph Firth

**Affiliations:** ^1^School of Health Sciences, Faculty of Biology, Medicine and Health, The University of Manchester, Manchester, United Kingdom; ^2^Manchester Institute for Collaborative Research on Ageing, Manchester, United Kingdom; ^3^Manchester Academic Health Science Centre, Manchester, United Kingdom; ^4^Manchester University NHS Foundation Trust, Manchester, United Kingdom; ^5^National Institute for Health and Care Research, Applied Research Collaboration Greater Manchester, School of Health Sciences, Faculty of Biology, Medicine and Health, The University of Manchester, Manchester, United Kingdom; ^6^Academic Collaborative Center Older Adults, Tilburg School of Social and Behavioral Sciences, Tilburg University, Tilburg, Netherlands; ^7^Community Therapy, Wigan and Leigh Teaching Hospitals NHS Foundation Trust, Manchester, United Kingdom; ^8^Acute Medicine and Elderly Care Department, Manchester University National Health Service Foundation Trust, Manchester, United Kingdom; ^9^Trauma and Orthopaedics Department, Trafford Hospital, Moorside Road, Manchester University National Health Service Foundation Trust, Manchester, United Kingdom; ^10^Division of Psychology and Mental Health, Academic Health Science Centre, The University of Manchester, Manchester, United Kingdom; ^11^Manchester Academic Health Science Centre, Greater Manchester Mental Health NHS Foundation Trust, Manchester, United Kingdom

**Keywords:** exergame, active video game, strength and balance, falls, fall prevention, older adults, sheltered housing, telecare

## Abstract

**Introduction:**

Falls prevention is a global priority given its substantial impact on older adults and cost to healthcare systems. Advances in telerehabilitation technology such as `exergaming’ show potential for delivering accessible, engaging exercise programs for older adults. This study aimed to establish the feasibility, acceptability and usability of exergaming in sheltered housing.

**Methods:**

A mixed-methods study with participants randomised in 2 sheltered housing facilities to intervention (*n* = 1 home, 12 participants) and control (*n* = 1 home 2, 12 participants) provided usual care for all, (physiotherapy prescribed strength and balance exercises and falls prevention advice) and a 6-week supervised exergaming programme (MIRA) offered 3 times per week to the intervention group only. At 6 weeks, feasibility, usability and acceptability outcomes were collected and analysed using descriptive statistics; qualitative focus groups with participants and interviews with staff were also completed and thematically analysed to elicit barriers and facilitators to usability and acceptability.

**Results:**

Mean exercise per week increased from 10.6 to 14.1 minutes in the control group and 9.6 to 36.8 minutes in the intervention group. All study processes and measures appeared feasible; 72% of those invited consented to taking part and 92% completed 6-week follow-up. Individual domains for the System Usability Scores (SUS) showed participants felt `very confident’ using the system with support (70%), would `like to use exergames frequently’ (50%) and found the system `easy to use’ (90%). However, they also felt they `needed to learn a lot at the beginning’ (40%) and would `need technical support’ (70%) for independent use of the exergames. Mean overall SUS was 63 reflecting moderate usability for independent use. Qualitative data indicated exergames were well received and highlighted motivational and social aspects; costs and set up. Staff also felt exergaming complemented traditional care.

**Discussion:**

Our study contributes to the evidence guiding the use of exergames to deliver suitable falls prevention interventions for older adults within sheltered housing in community settings.

## 1 Introduction

Aging population structure is an issue faced by nearly all societies across the world. For example, by 2050, the average age of the population is expected to increase by 30% in the United Kingdom, and 22% globally with more than a 75% increase in the proportion of those aged ≥65 years (1–3). Considering that per-capita healthcare costs for those aged ≥65 are over three-times higher than the general population (4), financing the provision of adequate care to the aging population is a major challenge (5). Nonetheless, capitalizing on novel technologies, and focusing on preventative care, are thought to be two potential solutions requiring further exploration (5, 6).

Within this broader issue, international health bodies have identified falls prevention as one key priority for tackling the health and social care challenges posed by aging population structure (7–9) due to the substantial personal and financial cost which falls impose on older adults. For instance, fall-related injuries are a leading cause of morbidity and mortality among older people (9), and account for a sizeable proportion of injury-related healthcare costs across the entire population (7), along with drastically reducing the quality of life for those affected (7–11).

One evidence-based intervention for falls prevention in older adults is multicomponent strength and balance training (8, 12); which is supported by top-tier evidence from systematic reviews and meta-analyses showing high clinical efficacy and good cost-effectiveness (12–14). However, many older adults do not have access to supervised strength and balance training, and difficulties in maintaining motivation for home-based exercise regimes poses a barrier toward long-term adherence (14–16). Thus, novel approaches toward the delivery of accessible and engaging exercise programs for older adults are urgently needed. World guidelines on falls prevention conditionally recommend using telehealth in combination with physical exercise as part of falls prevention programs but as yet, there is limited evidence on telehealth interventions in community settings (7).

A potential approach, made possible by advances in motion tracking technologies, is the use of ‘exergaming’ for delivering strength and balance training to older adults. The term ‘exergames’ refers to physically interactive video games; in which the player produces specific body movements to progress within the game, usually in response to set tasks or visual cues (17). Exergames can be used as a type of telehealth or telerehabilitation program for therapeutic purposes and were first popularized by commercial gaming systems, such as Nintendo’s ‘Wii’ console (with games such as ‘Wii Fit’ and ‘Wii Sports’) and Microsoft’s ‘Kinect’ system. However, beyond recreational uses, exergames have also been the subject of considerable research interest, collectively showing how exergames may provide an acceptable method for delivering therapeutic exercise training, with various clinical benefits. For instance, an emerging body of clinical trials have indicated that exergames can provide efficacious interventions for reducing childhood obesity, promoting rehabilitation in people with Parkinson’s disease, reducing depression, and improving cognitive functioning (17–22). Of particular relevance, a recent cluster-randomized controlled trial (C-RCT) in 106 older adults across 18 sheltered homes found that MIRA exergame training 3 times per week significantly reduced the incidence of falls compared to standard care (incidence rate 0.31, 95% CI 0.16 to 0.62, *p* = 0.001) over 12 weeks (mean total exergame time at 12 weeks = 359 min) (23). Additionally, the high cost-effectiveness observed within this trial further demonstrated the potential benefits of applying these new technologies toward falls prevention (23).

Given the indicated efficacy and cost-effectiveness of falls prevention exergames in controlled conditions further work is now required to establish the feasibility, usability and acceptability of such approaches. This is required to inform future uptake across sheltered housing facilities and further understand practical considerations of use within daily routines, training and support, and participant experiences.

## 2 Methods

Methods were informed by feasibility trials guidance (24).

### 2.1 Participants and procedures

The study was approved by the NHS Research Ethics Committee, United Kingdom (Ref15/WN/0047). Participants were recruited from two sheltered housing facilities in Manchester, England; with one home randomized by an independent statistician into the experimental group and one to the control group. Potentially suitable residential sheltered homes were identified through local housing association managers. For those interested in taking part, a meeting with the managers of these sheltered homes was arranged to acquaint them with the aims and details of the study, to gain their authorization and cooperation. When authorized by management, the research team, together with sheltered home managers, met with the residents to introduce the Exergames study to them. The sheltered home managers then identified residents who may meet inclusion criteria and ascertained if they were willing to be approached to receive information about the study. Potentially eligible residents were approached by the researcher to explain the study, answer any questions, provide written information sheets and consent forms, which were left with the residents for 24 h, before meeting in person to gain written, informed consent.

Consenting participants were assessed using the inclusion/exclusion criteria and screened by a trained research physiotherapist, before being formally admitted into the study. Inclusion criteria for the study were as follows: (i) sheltered home-dwelling residents aged 60 years and above; (ii) able to use exergaming technology safely as assessed by the therapist, with access to television and a 2 m space in the sheltered home to exercise safely; (iii) able to watch TV with or without glasses from a 2 m distance; (iv) English-speaking, and registered with primary care general practices; and (v) had mental capacity to give informed consent and comprehend the study procedures.

Exclusion criteria were (i) currently using gaming technologies for physical exercise; (ii) acute illness, severe congestive cardiac failure, uncontrolled hypertension, recent fracture or surgery; (iii) myocardial infarction or stroke in past 6 months; (iv) severe auditory, visual or cognitive impairment; (v) orthopedic surgery in last 6 months, or on waiting list to have orthopedic surgery; (vi) wheelchair users; and (vii) peripheral neuropathy or other uncontrolled medical conditions likely to compromise the ability to exercise.

Statistician-led randomization procedures were used to allocate the sheltered housing facilities to groups, i.e., treatment as usual (TAU) or TAU plus exergame intervention. Randomization occurred after baseline assessment, and the therapists performing the assessments were blinded to the participant group allocation. Participants were able to withdraw consent at any time without giving any reason and without their care or legal rights being affected.

### 2.2 Intervention and control conditions

#### 2.2.1 Control group

During the 6-week study participants in the control group were provided by a physiotherapist with standard community falls prevention advice, including the Age UK Staying Steady leaflet (25) and home exercises from the well-known OTAGO and FaME strength and balance program (26–28). For the 6 weeks of the study the control participants were asked by the physiotherapist to undertake three preselected exercises each week from the OTAGO program.

#### 2.2.2 Intervention group

**Figure 1:**
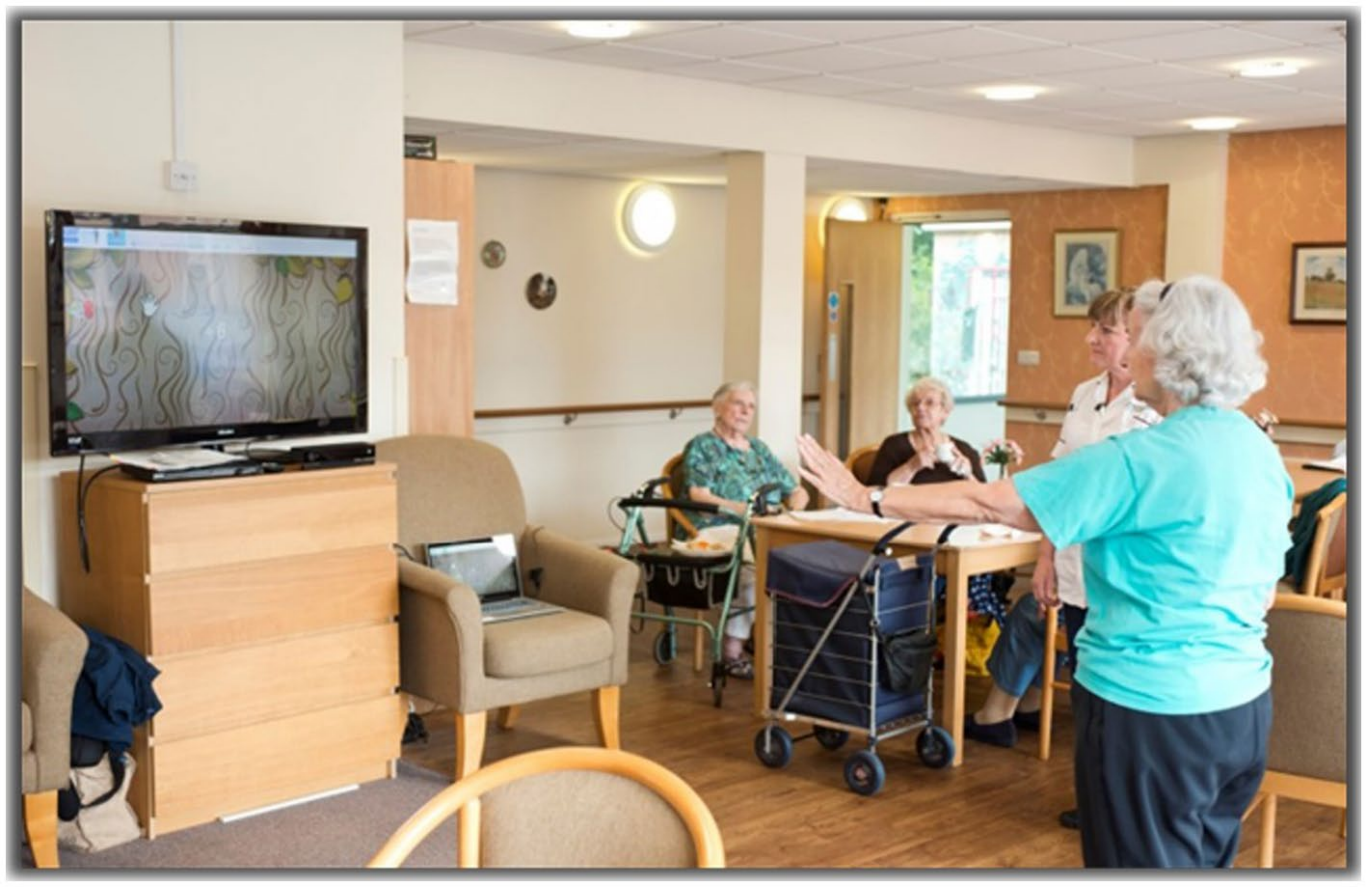
An example of a participant undertaking a MIRA Exergame session in the Sheltered Housing Facility.

**Figure 2:**
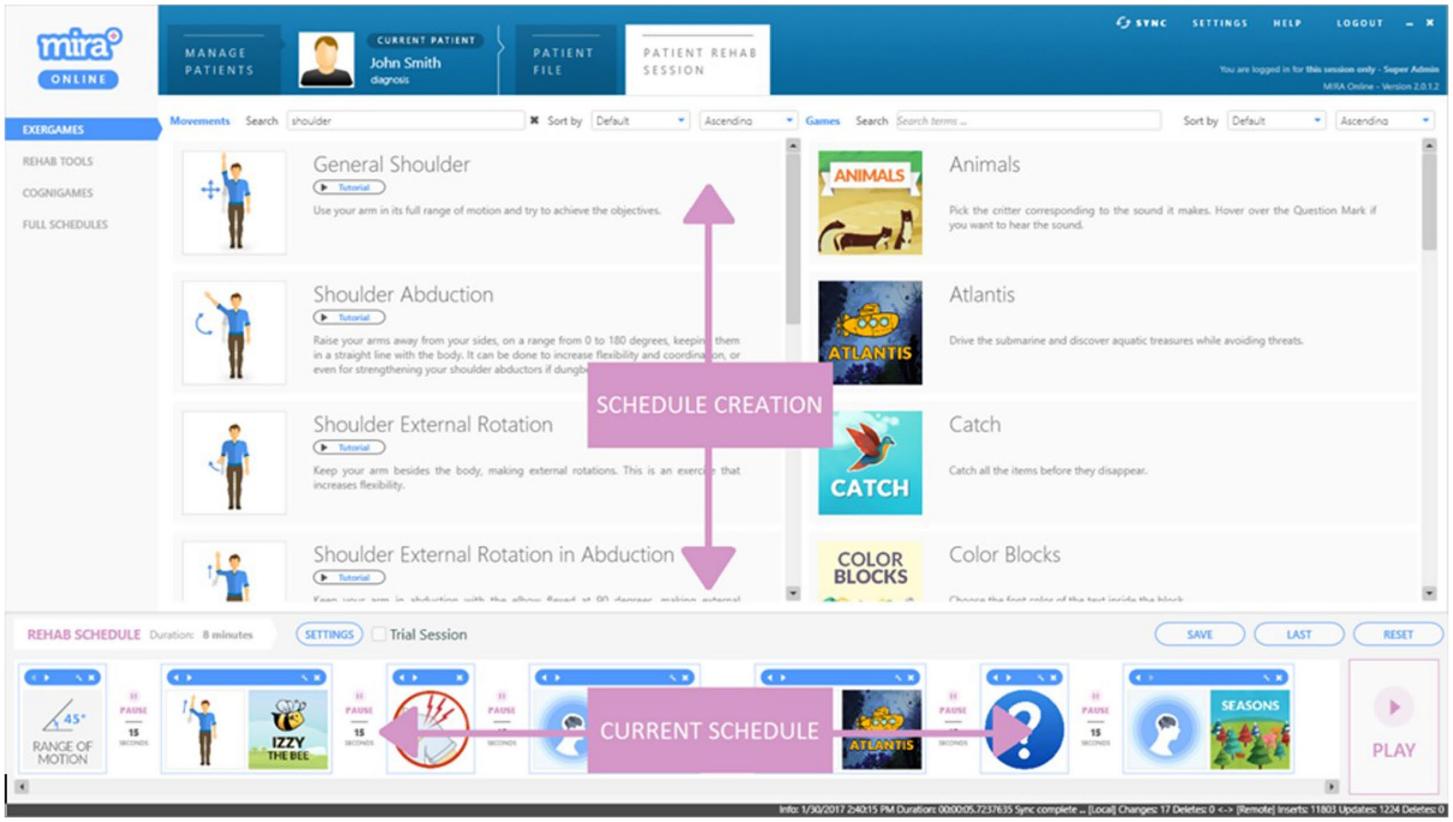
An example of a MIRA Exergame schedule with specific exercises and associated. Games (exercise-time per game and rest periods prescribed by Physiotherapist or other Practitioner) to create an individual program for each participant.

Participant in the intervention group undertook a 6 week exergame intervention in addition to the control group usual care. The MIRA rehab exergame program is a research-based Kinect exergame system, that can record validated patient measures (e.g., range of motion) and data on usage (frequency, duration). The MIRA exergames were developed with users, and based on best-practice strength and balance exercise [OTAGO (26, 27) and FaME (28)] currently used by therapists, and deemed safe for older people (i.e., low impact and joint protective). Specifically, the strength and balance exergames used in this study were developed to improve function, prevent falls and increase exercise adherence for older people in the sheltered home setting. Further information regarding MIRA exergames can be found on youtube video.^1^ Similar to the OTAGO and FaME exercise programs, the Exergames include a range of strength and balance exercises including knee flexors, knee extensors and hip abductors, ankle dorsiflexor and plantar flexor muscles all of which are important for strengthening and recovering balance. The MIRA exergame system also includes the capability to monitor real-time adherence (via motion tracking and detection) and progression, actively interacting with the user, and with motivational features such as point scoring and positive feedback (see Figures 1, 2).

Specifically, the intervention group were prescribed a program of standardized exergames sessions on a one-to-one basis three times per week for 6 weeks. The sessions took place under the supervision of a physiotherapist or physiotherapist assistant. The supervision of the participants included verbal instructions regarding how to perform correct movements and play the exergames in the initial sessions and progressed to observation of the participants once they were more familiar with the intervention after 2 or 3 sessions. The exergames sessions were situated in communal rooms in the assisted living facility. After an initial demonstration of the exergames, sessions were tailored to individual abilities and over the 6-week study gradually progressed, e.g., including more Exergames within a session, greater challenge, or longer duration. Exercises and games were tailored to participant’s ability in terms of duration and difficulty, which was determined by the physiotherapist. Individual exergames were undertaken independently by the intervention group participants (with the supervising therapists observing and able to act on and report any unexpected adverse events). Exergames were used to target different participant goals such as improving balance and strength. The patient’s performance was observed and reviewed at each session by the physiotherapy or physiotherapy assistant and the exercise schedule would be altered if the patient had improved. This included increasing the duration and/or frequency of exergames or the addition of more exergames to increase the challenge.

Physiotherapists and physiotherapist assistants received approximately 30 min of training on the use of the system, including how to set up the laptop and Kinect sensor (version 2; Microsoft Corp., Redmond, WA), and an introduction to the range of games and exercises which could be included in a participant’s individual program. After the 6 weeks of undertaking the strength and balance exercises both groups ceased, but in order to allow all participants to try the exergames, the control group participants were offered the opportunity to use the Exergame platform for 6 weeks. However, no trial data was collected from the control group during this period.

### 2.3 Feasibility assessments and quantitative outcomes

The main aims of the feasibility study were to evaluate the usability and acceptability of the exergame intervention; and the recruitment retention, and weekly attendance rates. Recruitment rate was calculated as the number of service users consented to the study, divided by the total number of service users invited to participate. Retention rates were examined as numbers of participants who undertook the weekly exergame training sessions, divided by the number originally enrolled in the study. Duration of exercise (minutes) was measured using the MIRA Exergame system. Alongside this, the System Usability Scale (SUS) (29) was administered at 6 weeks, to the intervention group only, to capture participant opinions on the usability of the exergame system in the context of sheltered housing. Acceptability of the exergames was explored through in-depth interviews and focus groups described in 2.4 below.

Assessments of specific outcome measures for functional ability, cognition and fall history were also undertaken to inform future pilot or clinical trials in exergames in sheltered home settings. These constructs were assessed at baseline and at 6 weeks using the following standardized tools, including:

- The Berg Balance Scale (BBS) to assess static and dynamic balance (30).
- Short Falls Efficacy Scale International (Short FES-I) to assess fear of falling (31).
- The Addenbrooke’s Cognitive Examination III (ACEIII) to assess cognitive function (32).
- The Physical Activity Scale for the Elderly (PASE) to assess user’s enjoyment of the exergames (33).

### 2.4 Qualitative data collection

To complement the quantitative measures, qualitative in-person interviews were undertaken with the sheltered home staff and a focus group with all the residents (*n* = 12) after 6 weeks use of the exergames, to gain further insights into the perceptions of the system, along with understanding the operations of the sheltered homes and how the exergames could best be incorporated into the residents’ routines.

Furthermore, qualitative interviews sought to assess participants’ experiences of the exergames, and identify the training and support required to facilitate the integration of the exergame technology into these settings.

### 2.5 Data analysis

Statistical analyses were conducted in SPSS (Version 25) (34). This was a feasibility study and therefore not powered to detect statistically significant differences. Feasibility measures were summarized with sample means, standard deviations of the means, and percentile scores where appropriate. Changes in secondary outcomes from pre-intervention to week 6 were analyzed using independent samples t-tests to calculate mean differences in change scores between exergame and control conditions (with 95% confidence intervals). Bonferroni-corrections for multiple testing were not applied to secondary outcomes due to the exploratory nature of these analyses.

The focus group and all interviews were digitally audio-recorded and transcribed verbatim. Qualitative data was managed using NVivo 12 software (35) and analyzed using the thematic analysis approach as described by Braun and Clarke (36). One researcher (CET) coded the participants’ responses and collated the codes into themes that were then corroborated by a second researcher (ES).

## 3 Results

### 3.1 Recruitment and retention

Across the two Sheltered Housing Facilities, a total of 33 individuals were approached and invited to participate in the study. Of these, 2 were ineligible due to severe cognitive impairment; 1 was registered blind, 1 was unable to attend due to other commitments, 2 were medically unstable, 2 used wheelchairs for all mobility needs and 1 had recently received orthopedic surgery. Therefore, 24 participants (72%) over a period of 4 weeks were enrolled in the trial (12 exergame condition, 12 controls).

Participants had a mean age of 74.3 years (range 54–91) and 83% were retirees (0 in paid employment). Whereas the control group were equally distributed for gender, 11/12 participants in the intervention group were female. The demographic characteristics of participants are displayed in Table 1. Scores on BBS, PASE, ACE-III, and FES-I items were similarly distributed between the two groups at baseline.

**Table 1:** Demographic characteristics of enrolled participants.

	Control group (*n* = 12)	Exergame group (*n* = 12)
Gender		
Females *N* (%)	6 (50.0)	11 (91.7)
Males *N* (%)	6 (50.0)	1 (8.3)
Age (years)		
Mean	70.4	78.2
SD	10.3	11.3
Range	59 to 91	54 to 91
Employment		
Retired *N* (%)	9 (75.0)	11 (91.7)
Doing voluntary work *N* (%)	1 (8.3)	0 (0.0)
Unemployed through sickness/disability *N* (%)	2 (16.7)	1 (8.3)
Marital status		
Single, never married *N* (%)	6 (54.5)	2 (16.7)
Married/living with partner *N* (%)	0 (0.0)	2 (16.7)
Divorced *N* (%)	2 (18.2)	0 (0.0)
Separated *N* (%)	1 (9.1)	0 (0.0)
Widowed *N* (%)	2 (18.2)	8 (66.7)

The retention and attendance rates at exergame sessions are displayed in Table 2. Over the 6 weeks, the mean number of participants attending the exergame group was 10.8 participants per week. In the exergame intervention group, 1 participant dropped out at week 3 and 1 dropped out at week 4, both giving reasons of feeling unwell. Of those retained in the study, non-attenders varied per session and reasons for non-attendance included hospital visits, other commitments such as family events and feeling unwell. No unexpected adverse events occurred during the study period.

**Table 2:** Weekly attendance^1^ and retention for exergame activities.

	Week 1 Exergames (*n* = 12)	Week 2 Exergames (*n* = 12)	Week 3 Exergames (*n* = 11)
Attendance Mean (S.D.)	0.75 (0.40)	0.66 (0.35)	0.62 (0.33)

	Week 4 Exergames (*n* = 10)	Week 5 Exergames (*n* = 10)	Week 6 Exergames (*n* = 10)
Attendance Mean (S.D.)	0.53 (0.32)	0.50 (0.32)	0.54 (0.37)

^1^Attendance means 3 sessions per week but participants attended fractions of a session, e.g., participants are coded as 0.a33/0.50/0.66 if they attended part of the sessions per week and 1 if they fully attended all 3 sessions per week. Reasons for participant drop out were due to becoming unwell and reasons for not attending sessions were due to other commitments such as hospital visits, family events.

### 3.2 Quantitative change measures in exergames and control conditions

All the measurement tools appeared feasible for use in the context of the cluster randomized trial of exergames in sheltered housing settings, with 92% of the participants (10/12 intervention; 12/12 control) completing the Short-FES-1, BBS, ACE-III, and PASE measures at baseline and 6-week follow-up. Changes in each of these measures in the exergame vs., control group are displayed in Table 3.

**Table 3:** Pre and post changes in outcome measures from Week 1–6 in exergame and control conditions.

	Control group (*n* = 12) PRE- measure mean (SD)	Control group (*n* = 12) POST- measure mean (SD)	Exergame group (*n* = 10) PRE- measure mean (SD)	Exergame group (*n* = 10) POST- measure mean (SD)	Control group (*n* = 12) 6 week mean change (SD)	Exergame group (*n* = 10) 6 week mean change (SD)	Mean difference (95% C.I.s)
Short Falls Efficacy Scale International (7–28) (Short FES-I)	18.3 (5.3)	18.3 (4.4)	16.4 (5.2)	15.0 (5.3)	0.00 (4.1)	−2.00 (3.3)	2.0 (−1.4 to 5.4)
The Berg Balance Scale (0–56) (BBS)	38.7 (12.2)	39.8 (10.8)	39.4 (11.3)	43.0 (11.9)	1.08 (7.9)	5.4 (6.13)	−4.3 (−10.7 to 2.1)
The Addenbrooke’s Cognitive Examination III (0–100) (ACEIII)	66.8 (19.5)	68.1 (20.0)	74.2 (15.8)	78.0 (17.2)	1.25 (5.9)	3.5 (6.9)	−2.3 (−7.9 to 3.4)
The Physical Activity Scale for the Elderly (0–400) (PASE).	68.7 (36.8)	66.6 (35.9)	76.5 (35.5)	92.5 (50.5)	−2.04 (23.3)	18.5 (37.3)	−20.6 (−49.6 to 8.4)

A notable observation is that the changes from Week 0 to Week 6 in each of the measures consistently favored the exergame conditions. For instance, mild reductions in fear of falling were observed following exergames (−2 points on FES-1) with no change in the control group. Small improvements in scores on balance and cognition measures observed in the control group were exceeded by improvements in the exergame group, which gained 4.3 more points on BBS scores and 2.3 more points on ACE-III scores by the 6-week follow-up. Alongside this, there was a slight reduction in self-reported physical activity in the control group (−2.04 on PASE) but a considerable increase in the exergames condition (+18.5 PASE). However, there were no significant differences between groups at any timepoint given the small sample size and moderate variance in measures (see Table 3). No adverse events were observed or reported during the study.

### 3.3 Participant evaluation of the intervention

Quantitative data on participant’s perceived usability of exergames are presented in Table 4; showing the results from the SUS at the 6-week follow-up for exergame group. Of note, 70% of the participants agreed or strongly agreed that they felt ‘very confident’ using the exergame system (SUS item 9), 40% reported they would like to use exergames frequently (SUS item 1) although 20% had no preference and 40% disagreed and 90% found the system ‘easy to use’ (SUS item 3). Although no participants found the system very cumbersome / awkward (SUS Item 8), 40% of participants found they needed to learn a lot at the beginning (SUS item 10), and 70% also felt they would need technical support for using the exergame system (SUS item 4) even though the physio set up the exergame system prior to each session.

**Table 4:** System usability scale results at 6-week follow-up.

Participants *N*=10^1^					
Level of agreement	1 - I think that I would like to use Exergames system frequently	2 - I found the system unnecessarily complex	3 - I thought the system was easy to use	4 - I think that I would need the support of a technical person to be able to use this Exergame system	5 - I found the various functions in this system were well integrated
[1] Strongly disagree *N* (%)	1 (10.0)	4 (40.0)	0 (0.0)	0 (0.0)	0 (0.0)
[2] Disagree *N* (%)	3 (30.0)	3 (30.0)	1 (10.0)	2 (20.0)	1 (10.0)
[3] No preference *N* (%)	2 (20.0)	2 (20.0)	0 (0.0)	1 (10.0)	2 (20.0)
[4] Agree *N* (%)	0 (0.0)	1 (10.0)	7 (70.0)	5 (50.0)	4 (40.0)
[5] Strongly agree *N* (%)	4 (40.0)	0 (0.0)	2 (20.0)	2 (20.0)	3 (30.0)
Mean (S.D.)	3.30 (1.567)	2.00 (1.054)	4.00 (0.816)	3.70 (1.059)	3.90 (0.994)

Level of agreement	6 - I thought there was too much inconsistency in the system	7 - I would imagine that most people would learn to use this system very quickly	8 - I found the system very cumbersome/awkward to use	9 - I felt very confident using this Exergame System	10 - I needed to learn a lot of things before I could get going with this system
[1] Strongly disagree *N* (%)	2 (20.0)	0 (0.0)	2 (20.0)	1 (10.0)	0 (0.0)
[2] Disagree *N* (%)	4 (40.0)	0 (0.0)	5 (50.0)	0 (0.0)	3 (30.0)
[3] No preference *N* (%)	1 (10.0)	4 (40.0)	3 (30.0)	2 (20.0)	2 (20.0)
[4] Agree *N* (%)	3 (30.0)	3 (30.0)	0 (0.0)	6 (60.0)	4 (40.0)
[5] Strongly agree *N* (%)	0 (0.0)	3 (30.0)	0 (0.0)	1 (10.0)	0 (0.0)
Mean (S.D.)	2.50 (1.179)	3.90 (0.876)	2.10 (0.738)	3.60 (1.075)	3.30(1.059)

^1^Two missing participants due to feeling unwell.

### 3.4 Qualitative data

These findings were reflected in the embedded qualitative study which obtained views and experiences, including perceptions, barriers and facilitators, around the implementation of exergames in shelter housing. Twelve residents who participated in the study attended a focus group and 3 staff (1 physiotherapist, 1 physio assistant and 1 sheltered housing manager) were interviewed individually.

#### 3.4.1 Perceptions of the exergame system

Overall, the exergames were well liked by participants, and staff were able to envision benefits of using the system. Participants were interested in finding out how the system worked and reflected on the potential health impacts of using exergames:

*“I think that to move the joints and muscles [as demonstrated] has got to be beneficial.”* (P3, resident focus group).

Although the games were designed to build on existing best practice strength and balance exercises, they were perceived to be new and different from other forms of exercise and this increased motivation for some participants:

*“I thought it’s very informative. Quite exciting some of the things you can do without too much exercise [physical exertion].”* (P2, resident focus group).

However, there was also an element of familiarity in some of the exercises which reminded participants of exercises they had undertaken in the past:

*“It’s about 70 years since I did them… I did them at school, but I’ve not done them since.”* (P8, resident focus group).

*“You better start now”* [the group laughs] (P9, resident focus group).

*“I am, I am now”* [the group laughs] (P8, resident focus group).

This aspect was also recognized by staff who suggested that ‘nostalgic’ elements may be motivating for participants:

*“I thought the yellow submarine was quite nice. I thought to the older generation, yellow submarine is something fun from their past* [which they could relate to]*… May-be the other different games could take up some cartoons from the past and build on that.”* (S99 staff interview).

One member of staff did suggest that one of the graphics looked too cartoonish, but this concern was not raised by any of the older participants.

Participants were positive about the opportunities to bring new exercises like the exergames into the sheltered housing, and several valued the convenience of being able to complete the exercises indoors. This reinforced their ambition to stay as healthy as possible:

*“Especially since you do not have to go outside…”* (P9, resident focus group).

*“Not on a cold day like this…”* (Moderator).

*“Yes.”* (P10, resident focus group).

*“You need to be as independent as you can be”* [as you age] (P11, resident focus group).

Staff also recognized that the perceived fun element of exergames could be motivating for participants, and particularly for those who may not enjoy traditional exercise:

*“I think it’s great fun. I thought it’s far more motivating* [to use these exergames] *than those who do conventional exercises, who may face more challenges. And it’s far more interactive, is not it?”* (S99, staff interview).

Generally, there was a high level of interest in the system and a discussion about whether the exergames could be installed and used on other devices such as an iPad/tablet or mobile phone took place, together with interest in finding out whether the software could be purchased and how much it might cost for them to continue.

A minority of participants expressed initial apprehension about using the system and suggested not all older people would be positive about its introduction due to being fearful of new technology. For some this was an enduring issue which could prove difficult to overcome and may require additional support:

*“I have this sort of phobia about these things. I have tried my best with computers, mobile phones, et cetera… And my first thoughts were that the talk leading up to the information was very good. But when I actually saw that there was computer* [involved]*… well what was she doing; why was she going up and down; what’s that …? So, I think you might get bit of resistance from old people who do not like mobile phones and things like that…”* (P4, resident focus group).

Nevertheless, others considered this apprehension to be an initial barrier which they could envisage surmounting as they became familiar with the technology:

*“You do not realise that… really it is shadowing you. And that you are standing up and down, which is only … whatever comes from that transfers onto the screen. But if you have not realised that for a start… and you start going up and down, you will not know what you are doing.”* (P5, resident focus group).

*“[*At first it was really like*] when you first got on the computer screen, and you go, ‘Well, what’s that?’ I do not know if I would ever [*be able*] to do that.”* (P6, resident focus group).

#### 3.4.2 Facilitators and barriers

##### 3.4.2.1 Incorporating exergames into the resident’s routine

Considerable discussion emerged when participants and staff were asked to consider where they thought the exergame system would be best placed within the sheltered housing to facilitate its use. There were differences of opinion around whether the system should be in a communal area, or in individual’s own room within the complex. Those who favored setting up the equipment in a shared space, thus allowing it to be used in a group session, suggested that the social aspect of completing the games in a group would be beneficial and motivational:

R35: *“I just think it’s fun to do it in a group as a social activity. I think you’d get to laugh and a laugh is as good as anything else.”* (P8, resident focus group).

and

*“I think we could do with a social activity in a setting like this, as some of them may not be getting enough socials...* [and their rooms may not be very conducive].” (Manager, staff interview).

Similarly, they felt that the exergame system in a communal area might encourage people to take part in the activity:

*“And as a group, it’ll be captive audience, will not it, really…”* (P13, resident focus group).

*“And if we work in a group, I think it would be beneficial really.”* (P14, resident focus group).

A number of participants found the element of competition against other people in the group compelling, although this is not one of the stated aims of the exergaming system.

Staff also suggested that positioning the exergames in a communal area would provide a focal point for potential users, and possibly encourage a wider range of people to take part:

*“I think there is an advantage in using a community room or activity room like this for these exergames… where people can use the rooms… so shared facility like this… maybe from a focus point of view, if you have got people* [enrolled], *then they could come into here to do the exergames. It would be like a clinic really.”* (Manager, staff interview).

Enabling free access to the system in a communal area was also considered to facilitate ongoing use and enable participants to fit the exercises more readily into their preferred routine, although this may impact on the social benefit of having organized times and sessions to complete the exergames as a group:

I think also they often like to do it if it’s spread out throughout the day like it’s part of their routine – like a daily routine. Yeah, that might help. It would help if they can come in and do it [throughout the day]. (S99, staff interview).

In contrast, other participants did not welcome the group aspect of placing the exergames in a communal area and identified this as a potential barrier. The thought of exercising in front of a group of spectators was unpalatable:

*“It might put some people off if you have to do it in front of other people… It’s a privacy matter.”* (P26, resident focus group).

Embarrassment and the thought that others in the group might comment or mock them while they were using the system were concerns, despite other participants offering reassurances that this would not happen:

*“When I said that, what I meant was that if there were other people there who were making silly remarks, et cetera, you’d like to prefer that not to happen.”* (P29, resident focus group).

*“I do not think they would though because it’s going to be their turn to get up and do it, is not it. We are all going to do it*… “(P30, resident focus group).

*“Yes, but nobody can decide how a person feels. It’s an individual thing… It’s an individual thing…”* (P31, resident focus group).

Notwithstanding this, there was recognition from some participants and staff that social discomfort might be an initial state which would diminish as they became more familiar and comfortable with the system:

*“I guess people might want to start doing it with other people once they have got the gist of it. They just do not want to make a fool of themselves to start with. But then once they have got the idea of it, then it would become more social.”* (Physio, staff interview).

The issue of sufficient space surfaced as both a facilitator and barrier to using the exergaming system in a communal area, primarily depending on the layout of the facilities:

*“There is not a lot of spaces in sheltered housing schemes…in the lounges and places like that to actually get it spread really… There are lots of furniture normally because the sheltered housing schemes are quite small… which is why I think you would facilitate them in their rooms… And also the screen is quite small… You know it’s brilliant here [in this room] where you have got a big screen. But if you are on a PC or something, you just want to be careful that [in some sheltered schemes or private homes] some of the people may not be able to see from that distance.”* (Physio, staff interview).

However, another staff member raised the issue that resident’s rooms were often also too small to house the equipment:

*“Yes, I definitely think it would work better in an environment like this* [communal area] *than in somebody’s home, because it would be very rare that somebody would be able to have a TV set up in their own home and stand back two meters from the screen, and have the space to be able to safely do the exercises… Most of our patients’ rooms have very limited space.”* (Manager, staff interview).

There were also some concerns from staff about placing the exergames in individual homes in part due to the hardware but also in terms of ensuring residents carried out the exercises correctly. Queries around who would set up and maintain the equipment, the length of time it may take to set up each session, and residents abilities to manage their own sessions were raised:

“Would the patients have access to changing things at home on how they would progress exercise, because it would be quite dangerous” (Physio, staff interview).

##### 3.4.2.2 Health and cognitive function

Issues relating to health and cognitive function were seen by some staff and residents as barriers to participating in the exergames. Physically, issues with eyesight and hearing and existing health conditions were felt to potentially impact on access and participants looked for reassurance that the exergames system would not exacerbate existing conditions, or lead to participants developing injuries:

*“I had a comment about it might be difficult for the hard of hearing to hear the computerized voice not from the volume point of view.*” (Physio, staff interview).

“*Maybe making it* [the voice] *a bit more human* [voice] *would help?”* (Manager, staff interview).

“*Yeah… It’s difficult if you cannot hear anyway, it is difficult… and the computerized voice is not normal in a way.”* (Physio, staff interview).

*“Can you just clarify for these people here that you would be asking health questions… you know, hip replacement, broken bones,* etc. *You would not be asked to do anything that would impair anything that’s already going wrong.”* (S99, staff interview).

Furthermore, cognitive abilities were considered a possible barrier to inclusion, although none of the participants in this feasibility study was cognitively impaired:

*“I thought that the concept might the difficult for some people to grasp if they have any cognitive difficulty. The automated mode is kind of like physically laying down instructions, whereas if we have a different way of explaining things to people that we are giving exercise or introducing activities to them,* [that might help]*… I thought that might pose a problem with some of the people that may be doing the exercises.”* (S99, staff interview).

Other staff thought these barriers may be overcome with sufficient initial support and training on the games and suggested that it wasn’t only people living with a cognitive impairment who could experience these issues:

*“Yes. Well, some people do have good cognitive abilities, but the whole gaming world, the gaming experience is just completely alien to them, and they might find that hard to grasp.”* (Manager, staff interview).

#### 3.4.3 Incorporating exergames into current strength and balance programs

Staff were positive about the role of exergaming either alongside existing rehabilitation programs or dovetailing around formal care in a range of scenarios:

*“Yes, yes. It may just be a good thing to do after like Physio intervention of sort to people going afterwards to their sites, so that people can continue their exercises after their clinical/Physio intervention…”* (Physio, staff interview).

*“These might be useful for people who aren’t necessarily going to come to our referral [service] to prevent them from kind of losing their agility and their stand… you know to try and encourage them to stay active. So this is going to possibly be used as a preventative measure, and as health promotion type activity, because it is not as specific being a Physio for treatment of a condition.”* (Physio, staff interview).

As there was a clear progression of levels throughout the games staff suggested this would be self- motivating, whereby participants could see themselves improving and moving through the levels:

*“I suppose the scoring [or points which the patients can see] will challenge them as well [Another group member interjects: “Yes – they’d like that”]. You know, we do the out-come measures right at the end. You know you can feedback to the patients. They see the scores, and [realise] they can improve… You can increase the difficulty level when they are actually seeing that they are getting better. I do not know how complex [the games might get], but I can imagine someone looking up at their scores [and wanting to try harder to improve it]” (Physio, staff focus group) “They’re quite motivated, aren’t they, when they see change [improvements] on a regular basis.”* (Physio, staff interview).

Furthermore, because the exergames were built on existing strength and balance programs staff were able to see the games as complementary to their own role, and able to provide additional training and support to participants to help them achieve their goals:

“*I suppose it has got elements of the Otago [exercise program], and it’s continuing what we are prescribing anyway”* (Physio, staff interview).

“*The other thing also is … because the movement is based on the types of things you do now anyway, should anybody come into therapy* [for treatment], *do not you think there would be … there could be considered to be the element of consistency, since actually what you are asking them to do is very similar to what they have done before, but you are asking them to do it in a more intensive and more challenging way. So the message that people get is consistent*” (Physio, staff interview).

## 4 Discussion

This is among the first studies to combine quantitative and qualitative data sources to evaluate Kinect-based exergames in sheltered housing for older adults. Our feasibility C-RCT compared weekly exergame sessions to control conditions across two different sheltered housing complexes, including 24 older adults. The quantitative data show the majority of residents found the exergames usable and some expressed interest in participating in exergame sessions on a regular basis. However, the need for specialized support and training in using the system was acknowledged by most residents (Table 4). The qualitative findings broadly agreed with the quantitative data, while also providing more fine-grain understanding into the specific factors which impact on engagement and implementation. Specifically, the social aspect of exergaming was welcomed by most participants, although there were some who felt they would be embarrassed to use the system in front of others. For some the competition element of exergaming was important, whether that be competition against themselves or competition with other people using the system, and this could be a strong motivating factor to enable progression of exercise intensity, continue using the system and improve their performance.

These findings also fit with previous research, which indicates that although falls prevention is certainly a priority for health services (7–9), this does not always act as a primary motivating factor for older adults – who actually engage in activities such as exercise and exergames for intrinsically-motivating reasons, e.g., enjoyment of the intervention itself (37). Collectively, this speaks to the importance of discovering and implementing novel ways to engage older adults in exercise interventions. By focusing on participant enjoyment, monitoring, progress and feedback, exergames can apply the principles of ‘gamification’ to falls prevention, in order to potentially produce greater levels of uptake and sustained engagement than achieved through typical physical activity interventions (38).

Interestingly, recent systematic reviews and meta-analyses have also identified other digital technologies (such as smartphones and internet interventions) as feasible and effective tools for health promotion and physical activity interventions in older adult populations (39). While the evidence is still nascent, exergames are now similarly emerging as a novel but promising strategy for providing evidence-based exercise interventions in healthcare settings for older adults (40, 41). Within this, exergames may confer certain advantages over other technologies due to their clear suitability for delivering strength and balance-based training; which is among the most efficacious and cost-effective interventions for falls prevention (7, 11, 12). Additionally, multiple clinical trials have indicated exergames can also produce other benefits; such as improving cognition and general functioning (21, 41–43). Although this feasibility study was not powered to detect significant differences between groups, there was some indication of benefit in the domains assessed (Table 2) in the exergame condition. Furthermore, a recent larger-scale C-RCT (23) of the same exergame technology assessed here found significant effects for balance and fear of falling.

## 5 Limitations and conclusions

Although this study aimed to be as inclusive as possible for all residents within the participating sheltered housing, a proportion of residents were unable to take part (due to either ineligibility or lack of interest). The sample size was small and the findings cannot be generalized to frail older adults who may not be able to undertake unsupervised programs or to older adults outside of the sheltered housing setting. The intervention arm mainly included older women (92%) and it is recommended that future studies include more older men to establish their views on the use of exergaming in sheltered housing facilities. The inability of this study to recruit all potential participants may shed further light on why exergame interventions are still not standard practice in sheltered housing settings, despite increasing evidence for their efficacy. Thus, further research on how to reach those older adults who appear ineligible, or are disinterested, for exergame interventions is required. Similarly, future studies should also assess and address disengagement from the intervention, to gain greater understanding for how those who do not adhere to exergames can be provided with other forms of falls prevention and health promotion initiatives.

A similar limitation is that in line with feasibility studies with a small sample size, exploratory outcomes were underpowered to demonstrate efficacy. We were also unable to blind assessors and the duration of the exergame was limited to only 6 weeks. Future larger, fully powered RCTs with blinding and at least 12 weeks intervention duration are required to test the effectiveness of exergames in a range of older participants and settings.

## 6 Conclusion

This study contributes to the growing body of literature which suggests exergames can provide a suitable falls prevention intervention for older adults. Within this, our qualitative findings provide further insights into the broader benefits of exergaming, and how participants can become more engaged with these interventions. As the evidence base continues to expand, and as older adults becoming increasingly accustomed with the use of digital technologies to support healthy aging (40), these findings may inform future C-RCTs and RCTs and the potential implementation of exergames within sheltered housing settings. Further research in this area should prioritize definitive trials in clinical rehabilitation settings with embedded process evaluations to inform possible future implementation in practice.

## Data availability statement

The raw data supporting the conclusions of this article will be made available by the authors, without undue reservation.

## Ethics statement

The studies involving humans were approved by NHS Research Ethics Committee, United Kingdom (Ref15/WN/0047). The studies were conducted in accordance with the local legislation and institutional requirements. The participants provided their written informed consent to participate in this study.

## Author contributions

ES: Conceptualization, Funding acquisition, Methodology, Writing – original draft, Writing – review & editing. CE-T: Formal analysis, Writing – original draft, Writing – review & editing. WM: Investigation, Writing – review & editing. KB: Investigation, Writing – review & editing. JC: Writing – review & editing. BR: Writing – review & editing. JF: Formal analysis, Writing – original draft, Writing – review & editing.

## Footnotes

^1^see video at https://www.youtube.com/watch?v=-v0Owkc0uGQ&t=2s
